# Magnetic resonance elastography demonstrates increased brain stiffness in normal pressure hydrocephalus

**DOI:** 10.1186/2045-8118-12-S1-O38

**Published:** 2015-09-18

**Authors:** John Huston, Avital Perry, Nikoo Fattahi, Arvin Arani, Fred Meyer, Richard Ehman

**Affiliations:** 1 Mayo Clinic, USA

## Introduction

Normal pressure hydrocephalus (NPH) is a potentially reversible cause of ventriculomegaly characterized by a classic triad of gait disturbance, cognitive impairment and urinary incontinence in older adults. Shunt tube placement is currently the mainstay therapy for NPH. However, unpredictable therapeutic responses as well as associated surgical complications necessitate better characterization of surgical candidates. The purpose of this study was to use MR Elastography (MRE) to determine brain tissue stiffness and outcomes after shunting in NPH patients compared with age and sex matched healthy controls.

## Methods

With IRB approval 10 patients (age range of 67-79 years) with NPH who were scheduled for ventriculoperitoneal shunting underwent preoperative MRE and were correlated with 21 age- and sex-matched normal controls (age range of 67-80 years). Studies were performed on a 3T scanner with a single-shot spin-echo EPI pulse sequence. Shear waves were introduced into the brain through an external source of vibration using a frequency of 60 Hz. We calculated the elasticity of different regions of interest (ROI) in the brain including the whole brain excluding the cerebellum (cerebrum), frontal, temporal, parietal, occipital lobes, deep grey matter/ white matter and the cerebellum. Associations between ROIs and symptoms were evaluated including cognitive decline, urinary incontinence, gait disturbance, duration of symptoms, opening pressure, improvement after lumbar puncture, and postoperative improvement. Statistical analysis included t test and linear regression.

## Results

MRE demonstrated significantly increased parenchymal stiffness among NPH patients as compared to normal controls in multiple ROIs, including the cerebrum, occipital, and parietal lobes (p=0.042, p=0.002, p=0.011 respectively). Postoperative improvement was associated with significant increased stiffness in deep grey/white matter, whereas postoperative failure was associated with significant increased temporal lobe stiffness (p=0.013, p=0.012).

## Conclusions

Brain MRE of patients with NPH revealed increased stiffness of the cerebrum, occipital and parietal lobes with a significant association between a range of NPH symptoms including the classic clinical triad with increased parenchymal stiffness. Surgically-responsive NPH was significantly associated with deep grey/white stiffness. MRE may guide selection of patients for shunting in the setting of equivocal diagnoses of NPH.
